# Musculoskeletal Symptoms among Stone, Sand and Gravel Mine Workers and Associations with Sociodemographic and Job-Related Factors

**DOI:** 10.3390/ijerph17103512

**Published:** 2020-05-18

**Authors:** Abdulrazak O. Balogun, Todd D. Smith

**Affiliations:** 1 Department of Environmental and Occupational Health, Indiana University School of Public Health–Bloomington, Bloomington, IN 47405, USA; abalogun@indiana.edu; 2 Department of Applied Health Science, Indiana University School of Public Health—Bloomington, Bloomington, IN 47405, USA

**Keywords:** musculoskeletal disorders, musculoskeletal symptoms, mining, ergonomics, job demands

## Abstract

Stone, sand and gravel mining (SSGM) constitutes the vast majority of mining operations in the United States. Despite musculoskeletal disorders being one of the most common occupational health problems across several industries, limited research has examined the extent of reported musculoskeletal symptoms or disorders among actively employed SSGM workers. To address this knowledge gap, cross sectional data were collected from 459 SSGM workers in the Midwestern United States to determine the prevalence of musculoskeletal symptoms. Sociodemographic and job-related factors were examined to identify possible risk factors in SSGM. Musculoskeletal symptoms of the low back (57%), neck (38%), shoulder (38%) and knee (39%) were highly prevalent among SSGM employees. The results, among other findings, showed that working more than 60 h a week increased the likelihood of musculoskeletal symptoms at the low back (OR: 4.7 95% CI: 1.9–11.5), neck (OR: 5.1, 95% CI: 2.2–11.8) and knee (OR: 4.5, 95% CI: 2.0–10.3). Working as a mechanic/maintenance worker increased the likelihood of low back (OR: 2.1, 95% CI: 1.1–4.2) and knee (OR: 2.2, 95% CI: 1.1–4.6) musculoskeletal symptoms. Intervention measures aimed at improving ergonomic hazard identification for various job tasks as well as administrative controls limiting hours worked may help reduce the burden of musculoskeletal problems in the SSGM industry.

## 1. Introduction

Musculoskeletal disorders (MSD) are some of the most common occupational health issues present in several industries. Risk factors associated with MSD and associated musculoskeletal symptoms (MSS) include repetitive work, working with hands above shoulder height or below knee height, carrying heavy loads and operating vibrating tools [1,2]. All of these factors, as well as others, are present in the mining industry. In 2018, MSD accounted for 272,780 nonfatal occupational injuries in the United States, majority of which were back, shoulder and knee injuries [3]. Several studies on MSS and MSD have revealed a high prevalence of back pain in mineworkers [4,5,6], which has been positively associated with whole-body vibration [7,8] commonly experienced by heavy equipment vehicle operators [9]. Time spent working has also been associated with MSS and MSD. Mine workers who perform high intensity tasks for 10–12 h are at risk of musculoskeletal problems [10]. Job tenure has also been linked to experience and hazard familiarity [11], which can aid in better risk perception and hazard identification [12].

Mining operations in the United States can generally be categorized into three sectors, which include metal and non-metal mining, coal mining and stone, sand and gravel mining (SSGM). The most current data indicates SSGM operations constitute almost 80% of all mining operations spanning 10,595 mining sites and 101,851 employees [13]. According to accident/injury/illness (AII) data from the Mine Safety and Health Administration (MSHA), the SSGM sector accounted for 29% of the total MSD-related nonfatal injuries and illnesses in the mining sector from 2009 to 2013 [14]. Analysis of the most recently published MSHA AII data [15] revealed that in 2018, 1885 nonfatal lost-time injuries occurred in the SSGM sector. The body parts most frequently injured were fingers (*n* = 377; 20.0%), back (*n* = 246; 13.1%), knees (*n* = 136; 7.2%) and shoulders (*n* = 130; 6.9%). Other recent research within the SSGM sector has shed light on the impact of another class of injuries associated with SSGM employees, which is slips, trips and falls (STFs). STFs are the second leading cause of non-fatal injury and illnesses among SSGM employees [16]. Between 2008 and 2017, the rate of nonfatal slips, trips and falls was 62 per 10,000 fulltime employees, resulting in 23,800 total days lost each year and an estimated cost burden of $17.5 million per year [17]. Recovery from STFs may contribute to musculoskeletal injury [14] and the potential also exists for individuals with MSD to become victims of STFs. An investigation into the prevalence of musculoskeletal symptoms and associated risk factors in SSGM operations will aid the identification of problem areas that could benefit from interventions. These interventions could have a positive impact in the reduction of MSD and associated injuries including STFs, thus reducing the burden associated with these injuries.

Data related to MSD prevalence within the SSGM industry has been obtained largely from AII data from MSHA. While this database comprises all mining operations within the United States, possible errors/bias in data reporting and coding by mining site supervisors and researchers could present challenges regarding the accuracy of findings [14,18]. In addition, the data being analyzed is collected based on reported injuries (e.g., sprains and strains) versus those with MSS. This could lead to an underestimation of the actual prevalence of those having MSD and associated MSS in the SSGM sector. To the authors’ knowledge, this is the first study to conduct a survey of active SSGM employees in the United States in order to assess the prevalence of MSS among this group. In a bid to identify possible risk factors associated with MSS in SSGM, the potential roles of age, job category and hours worked per week were also examined. This research addresses a national safety problem and seeks to benefit the SSGM industry by providing guidance on job demands and risk factors that need to be countered or controlled to prevent MSD and associated MSS.

## 2. Materials and Methods 

### 2.1. Participants

Cross-sectional data were collected from 459 full-time workers employed in the SSGM industry. Mining companies participating were small to medium-sized businesses. Participants completed surveys prior to or during breaks while completing Mine Safety and Health Administration (MSHA) annual refresher training with their employer or at a training facility in the Midwestern United States. Participation in the anonymous survey was voluntary and consent was obtained prior to participation. Institutional Review Board approval, as exempt status (IRB#1902635452), was granted by Indiana University–Bloomington for this study.

### 2.2. Measures

Sociodemographic and work-related characteristics including age, gender, educational status, job title, length of time spent on the current job and number of hours worked per week were collected in the survey. MSS prevalence was assessed using measures adapted from the validated Dutch Musculoskeletal Questionnaire (DMQ) [19]. Participants were asked if they had experienced any pain or discomfort in the last 12 months at nine different body regions (neck, upper back, low back, shoulders, elbows, hands/wrists, hips/thighs, knees and ankles/feet). Job titles were categorized into office/clerical/professional, maintenance/mechanics, equipment operators and laborers, moving/rubber tire equipment operators and supervisors. 

### 2.3. Data Analysis

Descriptive statistics delineating mean (and standard deviation) or percentage frequency values were used to analyze the prevalence of MSS across different body parts within the sample of workers, as well as across age groups and job categories. The four body parts most impacted within the sample of workers were neck, low back, shoulders and knees. Logistic regression was used to examine the relationships between age, job category, hours worked per week, time spent on the job and MSS across the four body parts. Models were adjusted for gender and body mass index. Odds ratios (ORs) and 95% confidence intervals (95% CIs) were calculated for each model. Probability values of < 0.05 were considered statistically significant. All analyses were performed using SPSS version 25.

## 3. Results

### 3.1. Sociodemographic Characteristics

As shown in Table 1, most participants were male (423, 93%) with females comprising 7% (31). Mean age of participants was 45 (*SD* = 14). A total of 41.3% (*n* = 188) had their highest educational level as high school/GED and about 29% (*n* = 130) had some college or technical or vocational training. Bachelor’s and master’s degree holders made up less than 11% of the entire group (8.6% and 1.8% respectively). Worker categories included office, clerical and professional personnel (*n* = 71), laborers and equipment operators (*n* = 125), moving/rubber tire equipment/vehicle operators (*n* = 82), maintenance/mechanics (*n* = 83) and supervisors (*n* = 51).

### 3.2. Prevalence 

**Figure 1:**
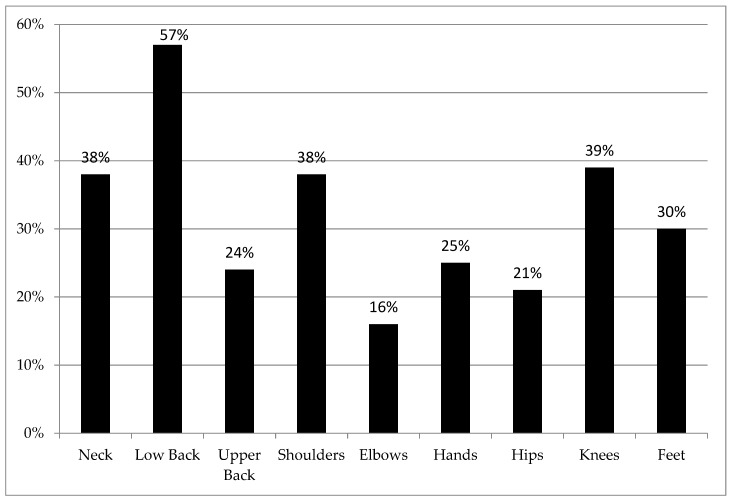
Percentage of stone, sand and gravel mining (SSGM) employees who experienced musculoskeletal symptoms (MSS) over the past 12 months on different body parts.

As observed in Figure 1, the low back was the most common body part employees reported experiencing MSS (57%) followed by knees (39%), neck and shoulders (38% each). MSS associated with the elbow had the lowest prevalence (16%).

Across all age groups and the nine body parts (Table 2), workers aged 35–44 had the highest MSS at five body parts (low back, upper back, shoulders, elbows and hands) while workers aged 45–54 reported the lowest prevalence at three body parts (low back, elbows and hands). Workers aged 35–44 reported the highest prevalence of low back MSS (69%) followed by workers aged 25–34 (61%) and workers aged 55–64 (60%). The lowest prevalence of 48% was observed in workers aged 45–54. For knee MSS, workers aged 65 and above reported the highest prevalence at 52% followed by workers aged 25–34 (44%) and workers aged less than 25 (43%). Workers aged 55–64 reported the lowest prevalence of knee MSS (32%).

Across job categories (Table 3), SSGM mechanics/maintenance workers reported the highest prevalence in five of the nine body parts (neck, hands, hips, knees and feet), while office/clerical personnel reported the lowest prevalence in seven of the nine body parts (low back, upper back, elbows, hands, hips, knees and feet). Low back MSS ranged from 49% (office or clerical personnel) to 71% (supervisors) and the prevalence of neck MSS ranged from 34% (stationary equipment operators and laborers) to 46% (mechanics/maintenance workers).

### 3.3. Risk Factors

In the series of logistic regression models with job category as the independent variable (Table 4), mechanics/maintenance workers had twice the odds of developing low back MSS (OR: 2.1, 95% CI: 1.1–4.2) and knee MSS (OR: 2.2, 95% CI: 1.1–4.6) compared to office/clerical/professional personnel, the reference group. Supervisors were also at higher risk of MSS at the low back (OR: 2.8, 95% CI: 1.3–6.2) compared to the reference group. Equipment operators and laborers had higher odds of knee MSS (OR: 2.0, 95% CI: 1.0–3.9) compared to the reference group.

With regard to the relationship of hours worked per week and MSS (Table 5), generally, employees who worked more than 40 h per week had higher odds of developing symptoms. Employees who worked more than 60 h a week had approximately five times the odds of developing low back MSS (OR: 4.7 95% CI: 1.9–11.5), knee MSS (OR: 4.5, 95% CI: 2.0–10.3) and neck MSS (OR: 5.1, 95% CI: 2.2–11.8) compared to those who worked at or below 40 h per week (reference group). Those who worked 51-60 h per week also had significantly higher odds of developing neck MSS (OR: 2.7, 95% CI: 1.3–5.3) compared to the reference group.

Age was not significantly associated with MSS at any of the four body parts, as is evident in Table 6. 

## 4. Discussion

This is a novel cross-sectional study exploring the prevalence of musculoskeletal symptoms among SSGM employees in the United Sates. Differences between males and females were not explored in this study given the small number of females in our sample. However, the logistic regression models were adjusted for gender as well as BMI, which is a risk factor for MSD [20]. Musculoskeletal symptoms were mostly associated with the low back, knees, shoulders and neck. This is consistent with prior research, as these four body parts are the most prevalent anatomical sites for musculoskeletal problems in other occupations [21,22,23,24]. More than half of respondents reported low back MSS. The 35–44 age group reported the highest prevalence while employees in the next older age group (45–54) reported the lowest prevalence, possibly indicating that age does not appear to play a significant role in low back pain etiology among our sample of stone, sand and gravel mine workers. The youngest age group of workers (<25 years old) reported the lowest prevalence only in one body part, the upper back. The oldest age group (aged 65+) reported the highest prevalence with elbow and knee injuries, and the lowest prevalence of neck injuries. It should be noted however that this age group has the lowest number of employees (*n* = 25), which may have affected the results observed. Indeed, the results of the logistic regression models, with age as the independent variable, showed no significant associations with MSS. This is consistent with previous research that found no associations between age and musculoskeletal problems to the low back [25], neck [26] and shoulder [27]. Despite these results, injury prevention among older workers remains important as these workers are more likely to have lost workdays when injured, are less likely to return to work, will take longer to rehabilitate and have greater absences from work [28].

Workers in the field typically complete physically demanding tasks associated with mining operations. This may help explain why office/clerical/professional personnel recorded the lowest prevalence of symptoms at almost all body parts. Mechanics and maintenance workers, who service fixed and mobile heavy equipment, reported the highest prevalence of MSS at five body parts including hands, hips, feet, neck and knees. These workers typically have to climb or crawl under heavy machinery to fix problems, twist and turn to reach machine components, and are exposed to vibration generated by tools and equipment they utilize during their work. Maintenance employees at SSGM operations tend to have the highest prevalence of musculoskeletal problems at most body parts according to MSHA accident/injury/illness data. Mechanics along with mobile equipment operators and laborers were most likely to be involved in nonfatal slips trips and fall incidents in the SSGM sector [16]. Supervisors were also associated with higher odds of developing low back injuries, which could be due to the previous years of mining-related work experience, typically a prerequisite to taking on a supervisory role.

With regard to hours worked per week, working above 40 h a week was associated with increased odds of MSS at the four body parts examined when compared to working at or below 40 h a week. The largest odds (4.5–5.1 times higher) were observed among those who worked over 60 h a week. Associations were significant with three of the four body parts examined. This finding is aligned with previous research showing that longer work hours is associated with increased musculoskeletal problems in nurses [29], physicians [30] and construction workers [31].

Although our findings are novel and important, some limitations should be considered when interpreting the findings associated with the present study. This study used a cross-sectional design, which restricts our ability to determine causality. Additionally, survey participants were drawn from SSGM employees in the Midwestern United States working at surface mines; therefore, the results may not be fully generalizable to some workers in other regions or those in underground SSGM operations. Nevertheless, our key findings are in line with previous studies related to MSS and MSD prevalence conducted in other high-risk occupations including mining, as well as the most recent non-fatal injury data reported within the SSGM sector. The results of the present study extend and better quantify what we know about musculoskeletal problems in the SSGM industry. 

The results of the present study provide direction for additional safety and ergonomics research within SSGM. Future research should include broader studies incorporating larger samples of mine workers across many regions in the United States and internationally. Additionally, a critical examination of specific job tasks associated with maintenance operations and equipment operations would be beneficial to better pinpoint risks. Specific task analyses and the application of ergonomic analysis tools such as the rapid entire body assessment (REBA) [32] or rapid upper limit assessment (RULA) [33] may provide insights into specific job postures or tasks that need to be adjusted or abated from certain job tasks. In addition to examining maintenance and equipment operations, additional studies are needed to explore why supervisors are at risk for low back musculoskeletal problems as they had the highest prevalence of low back MSS.

Intervention research targeting MSD risk factors could also be conducted to test the effectiveness of alternative strategies or equipment in mining operations with the goal of reducing the risk of MSD and their symptoms in the SSGM industry. These targeted risk factors should include those identified in this study as well as others found in other studies relevant to SSGM operations and mining such as heavy lifting, abnormal posture while performing work [2], STF exposures [16] and occurrence of prior injury [21].

Means to prevent musculoskeletal problems in the SSGM industry are needed. This research may guide some of those initiatives. One of the most prominent findings in this study was the impact of long work hours on musculoskeletal symptoms. Administrative controls should be incorporated into safety efforts to curtail musculoskeletal problems. Job rotation or enlargement may suffice but limiting shift hours and reducing or eliminating overtime for labor-intensive tasks may be needed within SSGM operations to reduce risks. With this though, human resource programs, including pay would have to be assessed and addressed to avoid burdening workers. Additionally, job design changes may be needed to maintain productivity.

Another way to reduce musculoskeletal problems in the SSGM industry could be through proper hazard identification by employees and management, which might be fostered through enhanced training that addresses hazards associated with SSGM operations [11]. Our findings suggest a focus on hazards related to the low back, knees, shoulders and neck. Additionally, along with hazard identification, risk tolerance and decision-making needs to be addressed. It has been indicated that most mining operations have programs in place to identify hazards but fail to mitigate hazards due to a high tolerance for risk; thus, employee and management decision-making with regard to hazard control needs to be incorporated into training efforts and safety management programs [34].

Although projects aimed at reducing injuries in the SSGM are ongoing [11], it would be beneficial to further incorporate ergonomics into such programs so employees are able to perform work in a safer manner, reducing the risk of developing MSD. Wearable technology, which uses sensors to detect ergonomically hazardous postures, alerting the wearer, have also been tested and validated for use in the construction industry [35] and may be applicable to SSGM operations. However, widespread use of this technology might still be far in the future and research needs to determine its applicability in the mining industry. Lastly, it appears that participatory ergonomic programs and initiatives with a Total Worker Health^®^ focus are gaining support among researchers and practitioners [36,37,38]. Although research is needed to determine effectiveness in the context of SSGM mining operations, these efforts may prove worthwhile in protecting and promoting SSGM worker health.

## 5. Conclusions

Our findings revealed that MSS especially those associated with the low back, neck, shoulders and knees are prevalent among SSGM employees. Working extended hours and working as a mechanic or maintenance worker were associated with greater likelihood of MSS. Office/clerical personnel had lower prevalence, but supervisors had greater odds of low back MSS. Intervention measures aimed at improving hazard identification for various job tasks as well as administrative controls limiting shift hours may help to reduce the burden of MSD, associated symptoms and other associated injuries such as STFs in the SSGM industry.

## Figures and Tables

**Table 1:** Descriptive statistics of respondents’ sociodemographic characteristics and job categories.

Variable	Mean (SD)/Frequency (%)
Age	45 (14)
Sex	
Male	423 (93%)
Female	31 (7%)
Education	
Some High School	43 (9.5%)
High School Graduate/GED	188 (41.3%)
Some College or Technical/Vocational Training	130 (28.6%)
Associate degree	44 (9.7%)
Bachelor’s degree	39 (8.6%)
Master’s degree	8 (1.8%)
Race	
African American/Black	4 (0.9%)
American Indian/Alaskan Native	1 (0.2%)
Asian/Asian American	1 (0.2%)
Hispanic, Latino/a/x	17 (3.7%)
White	426 (93.6%)
Job Category	
Office/Clerical/Professional	71 (15.6%)
Maintenance/Mechanics	81 (17.8%)
Laborers and Equipment Operators	125 (27.5%)
Moving/Rubber Tire Equipment/Vehicle Operators	82 (18.0%)
Supervisors	51 (11.2%)
Miscellaneous/Others	5 (1.1%)

**Table 2:** Prevalence of reported 12-month musculoskeletal symptoms (MSS) stratified by age group.

Age	Neck	Low Back	Upper Back	Shoulders	Elbows	Hands	Hips	Knees	Feet
<25 (*n* = 40)	13 (33%)	21 (53%)	8 (20%)	14 (35%)	6 (15%)	9 (23%)	10 (25%)	17 (43%)	12 (32%)
25–34 (*n* = 81)	30 (37%)	49 (61%)	19 (24%)	27 (34%)	16 (20%)	14 (18%)	13 (16%)	36 (44%)	30 (37%)
35–44 (*n* = 96)	39 (43%)	63 (69%)	28 (31%)	40 (43%)	19 (21%)	35 (38%)	19 (21%)	39 (42%)	28 (30%)
45–54 (*n* = 97)	33 (34%)	46 (48%)	20 (21%)	36 (38%)	9 (9%)	16 (17%)	19 (20%)	33 (34%)	25 (26%)
55–64 (*n* = 113)	51 (46%)	67 (60%)	30 (28%)	41 (37%)	17 (16%)	29 (26%)	28 (25%)	36 (32%)	28 (25%)
65+ (*n* = 25)	7 (28%)	14 (56%)	5 (22%)	10 (42%)	5 (20%)	7 (28%)	5 (20%)	13 (52%)	8 (32%)

**Table 3:** Distribution of reported 12-month MSS by job category.

Job Category	Neck	Low Back	Upper Back	Shoulders	Elbows	Hands	Hips	Knees	Feet
Office/Clerical Professional (*n* = 71)	27 (39%)	35 (49%)	11 (16%)	28 (41%)	8 (11%)	12 (17%)	12 (17%)	20 (28%)	19 (27%)
Maintenance/Mechanics (*n* = 81)	36 (46%)	52 (66%)	23 (30%)	33 (42%)	15 (19%)	30 (39%)	21 (27%)	36 (46%)	25 (32%)
Laborers and Equipment Operators (*n* = 125)	42 (34%)	70 (57%)	34 (28%)	41 (34%)	16 (13%)	36 (29%)	27 (22%)	52 (42%)	37 (30%)
Moving/Rubber Tire Equipment Operators (n = 82)	35 (43%)	49 (61%)	19 (24%)	29 (35%)	13 (16%)	18 (23%)	17 (21%)	32 (40%)	24 (30%)
Supervisors (*n* = 51)	22 (43%)	36 (71%)	15 (31%)	23 (45%)	11 (22%)	11 (22%)	11 (22%)	22 (43%)	15 (29%)

**Table 4:** Odds ratios of MSS by job category.

Job Category	Low Back OR	Knee OR	Shoulder OR	Neck OR
Office/Clerical/Professional	Ref	Ref	Ref	Ref
Maintenance/Mechanics	2.1 (1.1, 4.2) *	2.2 (1.1, 4.6) *	0.9 (0.5, 1.8)	1.4 (0.7, 2.8)
Laborers and Equipment Operators	1.6 (0.8, 2.9)	2.0 (1.0, 3.9) *	0.6 (0.3, 1.2)	0.9 (0.5, 1.8)
Moving/Rubber Tire Equipment Operators	1.7 (0.9, 3.4)	1.7 (0.8, 3.5)	0.7 (0.3, 1.3)	1.3 (0.6, 2.5)
Supervisors	2.8 (1.3, 6.2) *	2.0 (0.9, 4.4)	1 (0.5, 2.2)	1.3 (0.6, 2.8)

* *p* < 0.05.

**Table 5:** Odds ratios of MSS by hours worked per week.

Hours Worked Per Week	Low Back OR	Knees OR	Shoulder OR	Neck OR
Up to 40 (*n* = 64)	Ref	Ref	Ref	Ref
41–50 (*n* = 213)	1.7 (0.9, 3.0)	1.4 (0.7, 2.5)	1.3 (0.7, 2.5)	1.7 (0.9, 3.3)
51–60 (*n* = 128)	1.4 (0.7, 2.5)	1.1 (0.6, 2.2)	1.3 (0.6, 2.4)	2.7 (1.3, 5.3) **
More than 60 (*n* = 47)	4.7 (1.9, 11.5) **	4.5 (2.0, 10.3) **	1.6 (0.7, 3.6)	5.1 (2.2, 11.8) **

** *p* < 0.01.

**Table 6:** Odds ratios of MSS by age.

Age	Low Back OR	Knees OR	Shoulder OR	Neck OR
<25 (*n* = 40)	Ref	Ref	Ref	Ref
25–34 (*n* = 81)	1.4 (0.6, 3.0)	1.1 (0.5, 2.3)	1.0 (0.4, 2.3)	1.1 (0.5, 2.6)
35–44 (*n* = 96)	1.8 (0.3, 3.8)	1.0 (0.5, 2.2)	1.6 (0.7, 3.5)	1.5 (0.7, 3.3)
45–54 (*n* = 97)	0.8 (0.4, 1.7)	0.7 (0.3, 1.5)	1.3 (0.6, 2.7)	1.1 (0.5, 2.3)
55–64 (*n* = 113)	1.3 (0.6, 2.6)	0.6 (0.3, 1.3)	1.2 (0.6, 2.6)	1.6 (0.8, 3.5)
65+ (*n* = 25)	1.2 (0.4, 2.3)	1.3 (0.5, 3.7)	1.5 (0.5, 4.4)	0.8 (0.3, 2.5)
